# In Vitro Detection of Dental Root Fractures with Cone Beam Computed Tomography (CBCT)

**DOI:** 10.5812/iranjradiol.11485

**Published:** 2014-01-30

**Authors:** Erdogan Fisekcioglu, Semanur Dolekoglu, Mehmet Ilguy, Nilufer Ersan, Dilhan Ilguy

**Affiliations:** 1 Department of Dentomaxillofacial Radiology, Yeditepe University Faculty of Dentistry, Istanbul, Turkey

**Keywords:** Tooth Fractures, In Vitro, Cone-Beam Computed Tomography

## Abstract

**Background:**

Since the diagnosis of non-displaced longitudinal fractures present difficulties for the dentist, three-dimensional evaluation is necessary.

**Objectives:**

The aim of this study is to demonstrate the accuracy of cone beam computed tomography (CBCT) in detecting dental root fractures *in vitro*.

**Materials and Methods:**

An in vitro model consisting of 210 recently extracted human mandibular teeth was used. Root fractures were created by mechanical force. The teeth were placed randomly in the empty dental alveoli of a dry human mandible and 15 different dental arcs were created. Images were taken with a unit Iluma ultra cone-beam CT scanner (Imtec Corporation, Germany). Three dental radiologists separately evaluated the images.

**Results:**

According to the fracture types and fracture presence, there was an overall statistically significant agreement between the key and readings. Kappa values for intra observer agreement ranged between 0.705 and 0.804 indicating that each observer gave acceptable ratings for the type and presence of fractures.

**Conclusions:**

Detailed information about root fractures may be obtained using CBCT.

## 1. Background

Fractures of the maxillofacial region present difficulties for the dentist, especially when they are localized to dental and paradental structures. Common diagnostic aids for pulpal and periapical conditions are percussion, palpation, tooth mobility, coronal color changes, pulp sensitivity tests and radiographs (1).

The diagnosis of nondisplaced longitudinal fractures, such as cracks and vertical root fractures is a significant challenge in clinical practice (2). In fractures of the alveolar process, fractures of the root may be emerged and these fractures are frequently not detected. Fractures in the middle or apical third of the root of permanent teeth could be manually turned to the adequate position and immobilized. The incidence of pulpal necrosis rates are 20-24% and for this reason, prognosis is usually affirmative (3). The fracture lines can only be seen if the X-ray beam passes parallel to the fracture line (4-6). Superimposition of other structures further limits the sensitivity of radiographs for the detection of longitudinal fractures (7).

Cone beam computed tomography (CBCT) is a technique that produces 3-D digital imaging at reduced cost and less radiation for the patient than traditional computed tomography scans. Other advantages of CBCT are easier image acquisition, higher image accuracy, reduced artifacts, faster scan times, and greater cost-effectiveness (8-10). Studies have suggested that CBCT provides accurate and reliable linear measurements for reconstruction and imaging of dental and maxillofacial structures (11-13).

The X-ray area of interest is limited by the action of collimation of the CBCT primary X-ray beam. Limiting the irradiation field to fit the field of view (FOV) with a reduced exposure of set dosage to the patient in addition to an improved image quality due to reduced scattered radiation allows this function to provide dosage savings (8-11).

Previous studies evaluated the accuracy of the CBCT system compared with digital periapical radiographs in the detection of vertical root fracture (VRF) (14, 15). Many in vitro studies stated the accuracy of CBCT in evaluating root fractures (16-18). It has also been suggested to use CBCT to evaluate the maxillofacial region in trauma cases (19).

## 2. Objectives

The aim of this study is to demonstrate the accuracy of CBCT in detecting dental root fractures in vitro.

## 3. Materials and Methods

### 3.1. Materials and Preparation

The present study was approved by the Institutional Review Board of the faculty. In the present study, an in vitro model consisting of 284 recently extracted (due to periodontal disease and orthodontic indication) human mandibular teeth without any root fractures was used. The teeth had not undergone any root canal treatment and had no root resorption or anomaly. A dry human mandible obtained from the anatomy lab was used to mount the specimens. Thirty-five teeth that did not fit into the empty alveoli of the dry mandible were excluded. The entire sample was kept hydrated during the process except during fracture induction and radiographic scanning.

Root fractures were created by mechanical force. For induction of horizontal and oblique fractures, this force was applied using a hammer with the tooth placed on a soft foundation as described in a previous study (15).

**Figure 1.**
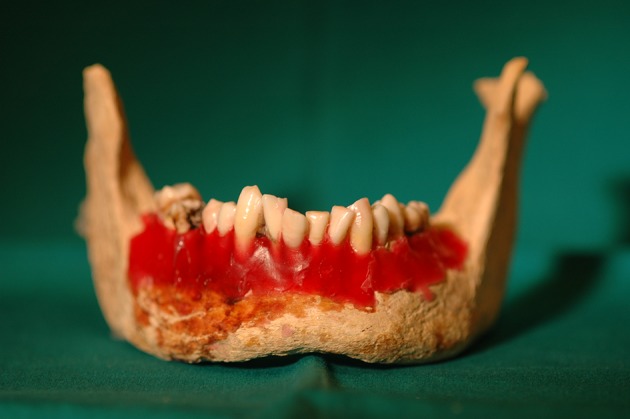
The teeth were placed randomly in the empty dental alveoli of the dry human mandible.

Vertical fractures on the other hand were created using a universal testing machine (Instron, Canton, MA, USA). The crowns of the teeth selected to be fractured were embedded in acrylic blocks to prevent splitting of the roots. The specimens were mounted on the lower plate of the universal testing machine and a compressive loading was applied vertically to the apex of roots with a loading rate of 1 mm/min until fracture occurred. Pilot studies were performed to determine the necessary force to break the root into two fragments. Then the two fragments of the teeth, which were completely separated, were relocated with super glue (Super Glue gel, 3M; Scotch, St Paul, MN, USA). After induction of fractures, 28 teeth that were separated into more than two fragments during fracture induction were excluded. Among the 221 remaining teeth, 210 were included in the study (60 molars, 60 premolars, 30 canines, and 60 incisors). The molars, premolars and incisors had 15 oblique, 15 horizontal and 15 vertical fractured teeth for each tooth type. Canines had 8 oblique, 8 horizontal and 7 vertical fractured teeth. The rest of the teeth remained intact without any induced fractures. The teeth were placed randomly in the empty dental alveoli of the previously mentioned dry human mandible and 15 different dental arcs were created to be scanned by a researcher. The dry mandible was covered with boxing wax to fix the teeth into the alveolar socket (Figure 1). 

**Figure 2.**
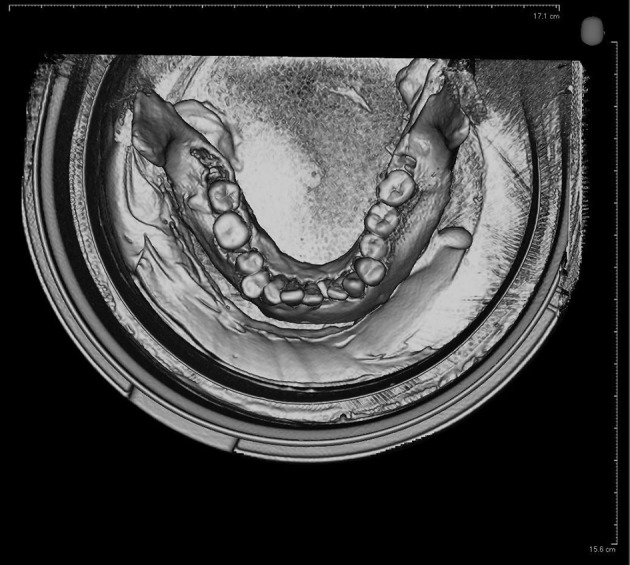
The 3D view of two cylindrical plastic holders, the smaller one fixed in the center of the other leaving 1 cm distance between them and filling the gap with water to mimic soft tissue.

There were two cylindrical plastic holders; the smaller one was fixed in the center of the other leaving 1cm distance between them and then to mimic the soft tissues, the gap was filled with water (Figure 2). 

### 3.2. Imaging Techniques

Digital images were taken with a large FOV Iluma ultra cone-beam CT scanner (Imtec Corporation, Germany) providing a 24.4 cm×19.5 cm amorphous silicon ﬂat-panel image detector and a cylindrical volume of reconstruction up to 21.1 cm×14.2 cm. Images were acquired at 120 kVp and 3.8 mA with a voxel size of 0.3 mm^3^. Axial scans and multiplanar reconstructions were obtained using the Iluma dental imaging software on a local workstation in accordance with the manufacturer’s instruction.

### 3.3. Image Assessment

Three calibrated dental radiologists separately evaluated the CBCT images for the presence of root fractures in a subdued room without knowing how many fractured teeth there were on each arc. The person who had fractured the roots and created the dental arcs, coded and randomized the CBCT images and prepared a chart for indicating the exact locations of each tooth to be used as a key at the end of the study. This researcher did not serve as an observer for detecting the fractures on the CBCT scans. Two different assessments were performed with a two-week interval. Only the research assistant knew the exact locations of fractured and intact teeth on each arc and he/she did not take part in the observation session.

**Figure 3.**
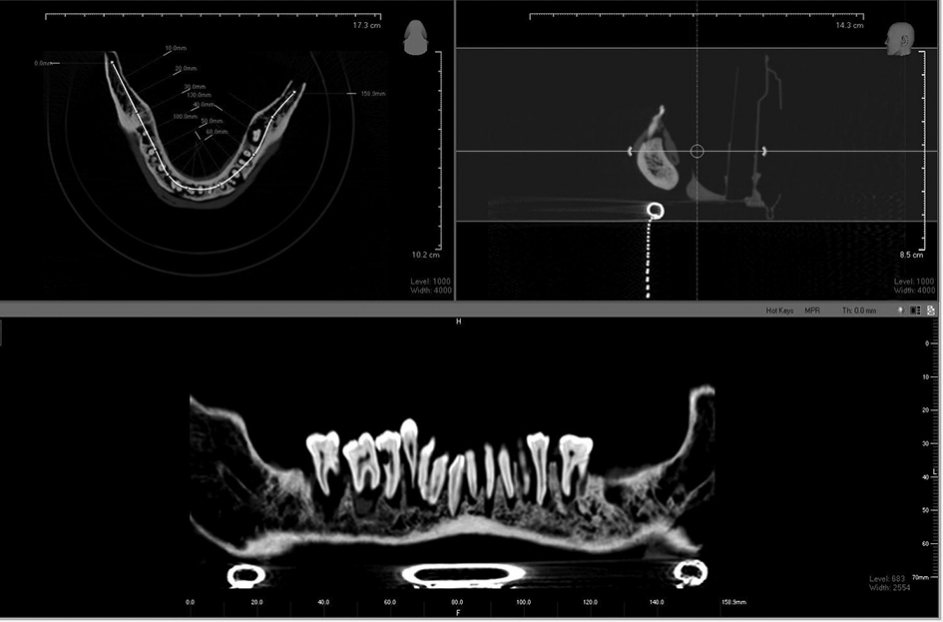
Panoramic reconstruction of CBCT images

**Figure 4.**
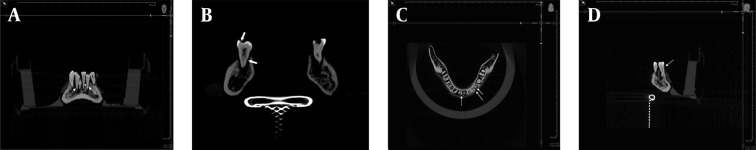
Iluma CBCT; A, Horizontal lines in the roots on the coronal scan show horizontal fracture. B, The radiolucent lines in the roots on the coronal scan; and C, the axial scan show vertical root fractures. D, When the fracture line is neither horizontal nor vertical on the sagittal plan, oblique root fracture is detected.

Radiolucent lines in the roots were regarded as fractures (Figure 3). When the fracture was neither horizontal nor vertical, it was evaluated as an oblique fracture (Figure 4). The time allocated for the observations was not restricted. The images were displayed and analyzed on a medical monitor. Adjustment of contrast and brightness could be done, if considered necessary, using the inbuilt image processing tools.

### 3.4. Statistical Analyses:

Sensitivity and speciﬁcity for each radiographic technique were calculated. Kappa statistics was used for assessing the agreement between observers. Chi-square statistics was used to determine whether there were differences between the systems. Results were considered signiﬁcant at P lower than 0.05.

## 4. Results

Considering the agreement between the key and the 1st and 2nd readings of each observer according to the fracture types and fracture presence, there was an overall statistically significant agreement between the key and readings (Tables 1 and 2). Kappa values for intraobserver agreement ranged between 0.71 and 0.86 indicating that the responses of the observers were close to each other for the type and presence of fractures (Table 3). Table 4 shows the calculated sensitivity, specificity, positive predictive value (PPV), and negative predictive values (NPV) of the first and second observations of each observer regarding the key. Interobserver agreements for determining the types and presence of root fractures can be seen on Table 5. 

**Table 1.** Agreement Between the Key and the 1st and 2nd Readings of Each Observer According to the Fracture Types

	Key No. (%)				Total No. (%)	Kappa	P-Value
	Non-Fracture	Vertical	Oblique	Horizontal			
**1st Observer 1st Reading No. (%)**						0.72	0.001
Non-fracture	46 (21.9)	7 (3.3)	4 (1.9)	10 (4.8)	67 (31.9)		
Vertical	1 (0.5)	33 (15.7)	1 (0.5)	2 (1.0)	37 (17.6)		
Oblique	3 (1.4)	6 (2.9)	46 (21.9)	0 (0.0)	55 (26.2)		
Horizontal	2 (1.0)	6 (2.9)	2 (1.0)	41 (19.5)	51 (24.3)		
Total No. (%)	52 (24.8)	52 (24.8)	53 (25.2)	53 (25.2)	210 (100)		
**1st Observer 2nd Reading No. (%)**						0.84	0.001
Non-fracture	52 (24.8)	4 (1.9)	6 (2.9)	6 (2.9)	68 (32.4)		
Vertical	0 (0.0)	42 (20.0)	1 (0.5)	2 (1.0)	45 (21.4)		
Oblique	0 (0.0)	2 (1.0)	45 (21.4)	0 (0.0)	47 (22.4)		
Horizontal	0 (0.0)	4 (1.9)	1 (0.5)	45 (21.4)	50 (23.8)		
Total No. (%)	52 (24.8)	52 (24.8)	53 (25.2)	53 (25.2)	210 (100)		
**2nd Observer 1st Reading No. (%)**						0.71	0.001
Non-fracture	52 (24.8)	6 (2.9)	16 (7.6)	8 (3.8)	82 (39.0)		
Vertical	0 (0.0)	41 (19.5)	2 (1.0)	1 (0.5)	44 (21.0)		
Oblique	0 (0.0)	2 (1.0)	30 (14.3)	3 (1.4)	35 (16.7)		
Horizontal	0(0.0)	3 (1.4)	5 (2.4)	41 (19.5)	49 (23.3)		
Total No. (%)	52 (24.8)	52 (24.8)	53 (25.2)	53 (25.2)	210 (100)		
**2nd Observer 2nd Reading No. (%)**						0.72	0.001
Non-fracture	52 (24.8)	5 (2.4)	12 (5.7)	11 (5.2)	80 (38.1)		
Vertical	0 (0.0)	42 (20.0)	1 (0.5)	3 (1.4)	46 (21.9)		
Oblique	0 (0.0)	3 (1.4)	36 (17.1)	3 (1.4)	42 (20.0)		
Horizontal	0 (0.0)	2 (1.0)	4 (1.9)	36 (17.1)	42 (20.0)		
Total No. (%)	52 (24.8)	52 (24.8)	53 (25.2)	53 (25.2)	210 (100.0)		
**3rd Observer 1st Reading No. (%)**						0.71	0.001
Non-fracture	49 (23.3)	12 (5.7)	7 (3.3)	8 (3.8)	76 (36.2)		
Vertical	0 (0)	33 (15.7)	0 (0)	0 (0)	33 (15.7)		
Oblique	2 (1.0)	5 (2.4)	41 (19.5)	4 (1.9)	52 (24.8)		
Horizontal	1 (0.5)	2 (1.0)	5 (2.4)	41 (19.5)	49 (23.3)		
Total No. (%)	52 (24.8)	52 (24.8)	53 (25.2)	5 (25.2)	210 (100.0)		
**3rd Observer 2nd Reading No. (%)**						0.73	0.001
Non-fracture	49 (23.3)	10 (4.8)	10 (4.8)	7 (3.3)	76 (36.2)		
Vertical	0 (0)	38 (18.1)	0 (0)	1 (0.5)	39 (18.6)		
Oblique	2 (1.0)	4 (1.9)	39 (18.6)	4 (1.9)	49 (23.3)		
Horizontal	1 (0.5)	0 (0)	4 (1.9)	41 (19.5)	46 (21.9)		
Total No. (%)	52 (24.8)	52 (24.8)	53 (25.2)	53 (25.2)	210 (100.0)		

**Table 2.** Agreement Between the Key and the 1st and 2nd Readings of Each Observer According to the Presence of Fracture

	TP ^a^	FP	FN	TN	Kappa	P-Value
**Key vs. 1st observer 1st reading**	137	6	21	46	0.69	0.001
**Key vs. 1st observer 2nd reading**	142	0	16	52	0.82	0.001
**Key vs. 2nd observer 1st reading**	128	0	30	52	0.68	0.001
**Key vs. 2nd observer 2nd reading**	130	0	28	52	0.70	0.001
**Key vs. 3rd observer 1st reading**	131	3	27	49	0.67	0.001
**Key vs. 3rd observer 2nd reading**	131	3	27	49	0.67	0.001

^a^Abbreviations: TP; True Positive, FP; False Positive, FN; False Negative, TN; True Negative

**Table 3.** Intraobserver Agreement for Determining the Types and Presence of Root Fractures

	Non-Fracture	Vertical	Oblique	Horizontal	Total No. (%)	Kappa	P-Value
	**1st Observer 1st Reading No. (%)**						
**1st observer 2nd reading No. (%)**						0.71	0.001
Non-fracture	54 (25.7)	3 (1.4)	6 (2.9)	5 (2.4)	68 (32.4)		
Vertical	4 (1.9)	31 (14.8)	5 (2.4)	5 (2.4)	45 (21.4)		
Oblique	2 (1.0)	1 (0.5)	41 (19.5)	3 (1.4)	47 (22.4)		
Horizontal	7 (3.3)	2(1.0)	3 (1.4)	38 (18.1)	50 (23.8)		
Total No. (%)	67 (31.9)	37 (17.6)	55 (26.2)	51 (24.3)	210 (100.0)		
	**2nd Observer 1st reading**						
**2nd Observer 2nd reading No. (%)**						0.86	0.001
Non-fracture	75 (35.7)	0 (0.0)	1 (0.5)	4 (1.9)	80 (38.1)		
Vertical	1 (0.5)	40 (19.0)	0 (0.0)	5 (2.4)	46 (21.9)		
Oblique	5 (2.4)	3 (1.4)	34 (16.2)	0 (0.0)	42 (20.0)		
Horizontal	1 (0.5)	1 (0.5)	0(0.0)	40 (19.0)	42 (20.0)		
Total No. (%)	82 (39.0)	44 (21.0)	35 (16.7)	49 (23.3)	210(100.0)		
	**3rd Observer 1st reading**						
**3rd Observer 2nd reading No. (%)**						0.84	0.001
Non-fracture	70 (33.3)	2 (1.0)	4 (1.9)	0 (0.0)	76 (36.2)		
Vertical	5 (2.4)	30 (14.3)	2 (1.0)	2 (1.0)	39 (18.6)		
Oblique	1 (0.5)	1 (0.5)	4 (20.5)	4 (1.9)	49 (23.3)		
Horizontal	0 (0.0)	0 (0.0)	3 (1.4)	43 (20.5)	46 (21.9)		
Total No. (%)	76 (36.2)	33 (15.7)	5 (24.8)	49 (23.3)	21 (100.0)		

**Table 4.** Diagnostic Efficacy of Different Observers’ Readings Versus Key in Diagnosing the Presence of Fracture

	Sensitivity (95% CI ^a^)	Specificity (95% CI)	PPV (95% CI)	NPV (95% CI)	PLR (95% CI)	NLR (95% CI)	Kappa (95% CI)
**1st observer**							
1st reading	0.87 (0.80-0.91)	0.88 (0.76-0.95)	0.96 (0.91-0.98)	0.69 (0.56-0.79)	7.51 (3.53-15.99)	0.15 (0.10-0.22)	0.66 (0.58-0.79)
2nd reading	0.90 (0.84-0.94)	1 (0.91-1)	1 (0.97-1)	0.76 (0.64-0.86)	-	0.10 (0.06-0.16)	0.81 (0.73-0.90)
**2nd observer**							
1st reading	0.81 ( 0.74-0.87)	1 (0.91-1)	1 (0.96-1)	0.63 (0.52-0.74)	-	0.19 (0.14-0.26)	0.68 (0.58-0.78)
2nd reading	0.82 (0.75-0.88)	1 (0.91-1)	1 (0.96-1)	0.65 (0.53-0.75)	-	0.18 (0.13-0.25)	0.70 (0.60-0.80)
**3rd observer**							
1st reading	0.83 (0.76-0.88)	0.94 (0.83-0.99)	0.98 (0.93-0.99)	0.64 (0.53-0.75)	14.37 (4.78-43.21)	0.18 (0.13-0.26)	0.67 (0.56-0.77)
2nd reading	0.83 (0.76-0.88)	0.94 (0.83-0.99)	0.98 (0.93-0.99)	0.64 (0.53-0.75)	14.37 (4.78-43.21)	0.18 (0.13-0.26)	0.67 (0.56-0.77)

^a^ Confidence interval

**Table 5.** Interobserver Agreement for Determining the Types and Presence of Root Fractures

	Non-fracture	Vertical	Oblique	Horizontal	Total No. (%)	Kappa	P-Value
	**1st Observer 1st Reading No. (%)**						
**2nd Observer 1st reading No. (%)**						0.57	0.001
Non-fracture	55 (26.2)	6 (2.9)	16 (7.6)	5 (2.4)	82 (39.0)		
Vertical	6 (2.9)	25 (11.9)	7 (3.3)	6 (2.9)	44 (21.0)		
Oblique	1 (0.5)	3 (1.4)	27 (12.9)	4 (1.9)	35 (16.7)		
Horizontal	5 (2.4)	3 (1.4)	5 (2.4)	36 (17.1)	49 (23.3)		
Total No. (%)	67 (31.9)	37 (17.6)	55 (26.2)	51 (24.3)	210 (100.0)		
**3rd Observer 1st reading No. (%)**							
Non-fracture	48 (22.9)	7 (3.3)	10 (4.8)	11 (5.2)	76 (36.2)	0.53	0.001
Vertical	4 (1.9)	20 (9.5)	5 (2.4)	4 (1.9)	33 (15.7)		
Oblique	4 (1.9)	7 (3.3)	37 (17.6)	4 (1.9)	52 (24.8)		
Horizontal	11 (5.2)	3 (1.4)	3 (1.4)	32 (15.2)	49 (23.3)		
Total No. (%)	67 (31.9)	37 (17.6)	55 (26.2)	51 (24.3)	210 (100.0)		
	**2nd Observer 1st Reading No. (%)**						
**3rd Observer 1st reading No. (%)**						0.53	0.001
Non-fracture	53 (25.2)	9 (4.3)	5 (2,4)	9 (4,3)	76 (36,2)		
Vertical	4 (1.9)	27 (12.9)	1 (0.5)	1 (0.5)	33 (15.7)		
Oblique	18 (8.6)	5 (2.4)	24 (11.4)	5 (2.4)	52 (24.8)		
Horizontal	7 (3.3)	3 (1.4)	5 (2.4)	34 (16.2)	49 (23.3)		
Total No. (%)	82 (39.0)	44 (21.0)	35 (16.7)	49 (23.3)	210 (100.0)		
	**1st Observer 2nd Reading No. (%)**						
**2nd Observer 2nd reading No. (%)**						0.72	0.001
Non-fracture	63 (30.0)	3 (1.4)	7 (3.3)	7 (3.3)	80 (38.1)		
Vertical	1 (0.5)	36 (17.1)	2 (1.0)	7 (3.3)	46 (21.9)		
Oblique	3 (1.4)	3 (1.4)	34 (16.2)	2 (1.0)	42 (20.0)		
Horizontal	1 (0.5)	3 (1.4)	4 (1.9)	34 (16.2)	42 (20.0)		
Total No. (%)	68 (32.4)	45 (21.4)	47 (22.4)	50 (23.8)	210 (100.0)		
**3rd Observer 2nd reading No. (%)**						0.65	0.001
Non-fracture	55 (26.2)	7 (3.3)	8 (3.8)	6 (2.9)	76 (36.2)		
Vertical	1 (0.5)	32 (15.2)	1 (0.5)	5 (2.4)	39 (18.6)		
Oblique	6 (2.9)	4 (1.9)	34 (16.2)	5 (2.4)	49 (23.3)		
Horizontal	6 (2.9)	2 (1.0)	4 (1.9)	34 (16.2)	46 (21.9)		
Total No. (%)	68 (32.4)	45 (21.4)	47 (22.4)	50 (23.8)	210 (100.0)		
	**2nd Observer 2nd Reading No. (%)**						
**3rd Observer 2nd reading No. (%)**							
Non-fracture	56 (26.7)	6 (2.9)	8 (3.8)	6 (2.9)	76 (36.2)	0.55	0.001
Vertical	3 (1.4)	32 (15.2)	2 (1.0)	2 (1.0)	39 (18.6)		
Oblique	11 (5.2)	5 (2.4)	26 (12.4)	7 (3.3)	49 (23.3)		
Horizontal	10 (4.8)	3 (1.4)	6 (2.9)	27 (12.9)	46 (21.9)		
Total No. (%)	80 (38.1)	46 (21.9)	42 (20.0)	42 (20.0)	210 (100.0)		

## 5. Discussion

Correct diagnosis of root fractures is of fundamental importance in dental practice. Conventional two dimensional radiography, including periapical and bitewing radiographs, is the current standard of detection of vertical root fractures (VRFs). This radiographic technique; however, is limited by its two dimensional nature, yielding an inability to accurately detect fractures that do not parallel the X-ray beam. There are previous studies reporting low values on conventional dental radiographs in the assessment of root fractures (20, 21). CBCT appears to be more accurate than conventional dental radiography in the detection of these occurrences (14, 22, 23).

In the recent years, CBCT scanners have been increasingly developed specifically for dental and maxillofacial imaging (9, 24-27). Overall, there are studies favoring the use of CBCT over conventional periapical (PA) radiograph. A study by Hassan et al. (22) found that CBCT had a significantly higher accuracy than PA radiographs. Kamburoglu et al. (28) showed that high resolution CBCT technology had a higher receiver operating characteristic curves Az value than digital PA radiograph. The disadvantages of CBCT imaging are inadequate soft tissue view and artifacts (9, 25). Inadequate soft tissue view may not be a problem in dentomaxillofacial imaging, because the teeth and bones are mineralized tissues. CBCT presents more accurate results than periapical radiographs in the diagnosis of horizontal root fractures, because it enables direct visualization of the fracture lines (16, 20, 29). CBCT systems are categorized as follows: small volume, usually used for scanning just a few teeth or one jaw; medium volume, covering both jaws, the maxillary sinus, and part of the nose; and large volume, covering the entire maxillofacial region, extending upward in some systems to the cranial vertex (30). In the present study, a large field of view (FOV) was used to detect root fractures based on the recommendation of the American Academy of Oral and Maxillofacial Radiology mentioning that the clinicians should evaluate the entire image volume acquired through the tomographic exam (31).

It is very hard to detect vertical and oblique root fractures unless the fragments are separated. Identification of vertical root fractures is almost impossible in some cases. VRFs and oblique fractures in non-endodontically treated teeth begin at the root apex and occur primarily in the buccolingual direction. Symptoms are usually minimal or absent in the early stages (31).

In the diagnosis of VRFs, usage of CBCT may be useful as the object could be visualized from different angles with very thin slices without disturbance of overlapping of structures (14, 22, 23, 28-32).

Da Silveira et al. (32) reported that the specificity, sensitivity and accuracy findings in detecting VRFs were significantly high for teeth that are not root filled. In this in vitro study, they found that the specificity, sensitivity and accuracy of CBCT were respectively 1, 0.97 and 0.98. Kajan et al. (33) stated that CBCT may be helpful in the assessment of the pattern of a fracture line and can help dentists to plan the treatment. The present study confirms the accuracy of using CBCT to diagnose root fractures and their patterns similar to these studies. The specificity, sensitivity and the PPV of CBCT were respectively 0.96, 0.84 and 0.98.

Radiographic detection of horizontal root fractures with CBCT is easier than that of VRFs that it is mainly seen in teeth with root canal treatment and posts that caused artifacts (17). Many studies reported that small FOV found higher levels of accuracy in detecting horizontal root fractures (18, 29). Consistently, Costa et al. (34) reported that it was difficult to diagnose horizontal root fractures through a large-volume CBCT by using a small voxel reconstruction. In traumatic cases, CBCT should be used in order to detect all possible fractures in the maxillofacial region. For this reason, we preferred to use large FOV in detecting horizontal and also vertical-oblique root fractures. Even though the observers reevaluated the fractures 14 days after the first observation and Kappa values show acceptable interobserver agreement, they were not very high. This indicates that there are still horizontal root fractures that could not be detected by any of the observers though most of them are easily identified with conventional two-dimensional radiographic procedures. This may be due to the smaller resolution of the image in large-volume CBCT that might have negatively affected the diagnosis of horizontal root fractures. Even when there is no time limitation and all sections can be interpreted, fracture lines might be overlooked. Especially in traumatic cases, as the condition needs urgent therapy, it is impossible to evaluate the fractures in 15-day intervals. With time, a traumatized patient could come to the clinic with dull pain on mastication as a result of the separation of the fractured root segments (35, 36). In this situation, the raw CBCT data of the patient may be reevaluated for assessment of root fractures by a maxillofacial radiologist regarding the “as low as reasonably achievable” (ALARA) principle. Since clinical findings are important, the dentomaxillofacial radiologist should be able to make the clinical examination of trauma patients. Although posttraumatic pain is generally accepted as normal, it might as well be an indicative of the presence of root fracture. Many variables such as beam angulation, exposure time, receptor sensitivity, processing viewing conditions, superimposition of anatomical structures and localization and type of fractures are factors that influence the clinical diagnostic capacity of intraoral radiography. Therefore, CBCT may be a beneficial addition to intraoral imaging in trauma cases. In trauma patients, the radiologist should examine the entire head for bone fractures as well as dental root fractures. Detailed information about root fractures may be obtained using CBCT and root fractures should be taken into consideration by radiologists as a possible occurrence and care must be taken to make a true diagnosis.
